# Linking Data for Mothers and Babies in De-Identified Electronic Health Data

**DOI:** 10.1371/journal.pone.0164667

**Published:** 2016-10-20

**Authors:** Katie Harron, Ruth Gilbert, David Cromwell, Jan van der Meulen

**Affiliations:** 1 Department of Health Services Research and Policy, London School of Hygiene and Tropical Medicine, 15-17 Tavistock Place, London, United Kingdom; 2 Institute of Child Health, University College London, 30 Guilford Street, London, United Kingdom; Stellenbosch University, SOUTH AFRICA

## Abstract

**Objective:**

Linkage of longitudinal administrative data for mothers and babies supports research and service evaluation in several populations around the world. We established a linked mother-baby cohort using pseudonymised, population-level data for England.

**Design and Setting:**

Retrospective linkage study using electronic hospital records of mothers and babies admitted to NHS hospitals in England, captured in Hospital Episode Statistics between April 2001 and March 2013.

**Results:**

Of 672,955 baby records in 2012/13, 280,470 (42%) linked deterministically to a maternal record using hospital, GP practice, maternal age, birthweight, gestation, birth order and sex. A further 380,164 (56%) records linked using probabilistic methods incorporating additional variables that could differ between mother/baby records (admission dates, ethnicity, 3/4-character postcode district) or that include missing values (delivery variables). The false-match rate was estimated at 0.15% using synthetic data. Data quality improved over time: for 2001/02, 91% of baby records were linked (holding the estimated false-match rate at 0.15%). The linked cohort was representative of national distributions of gender, gestation, birth weight and maternal age, and captured approximately 97% of births in England.

**Conclusion:**

Probabilistic linkage of maternal and baby healthcare characteristics offers an efficient way to enrich maternity data, improve data quality, and create longitudinal cohorts for research and service evaluation. This approach could be extended to linkage of other datasets that have non-disclosive characteristics in common.

## Introduction

Linkage of administrative or electronic health records for mothers and babies has the potential to provide a population-level resource to support research and service evaluation. Such linked data are increasingly used in populations around the world, including Scotland, Canada, Australia, the US and the Netherlands amongst others.[1–4] Linkage of primary care electronic health records for mothers and babies has been attempted for small populations in England [5, 6] and linkage of prospective maternity and children’s health services datasets on a larger scale is being developed by the Health and Social Care Information Centre (HSCIC).[7] However, routine linkage of maternal and baby records in existing administrative hospital data does not currently exist in England.

The large sample size and representativeness of linked administrative hospital data offer a cost-effective alternative to traditional cohort studies for studying childhood outcomes, providing valuable information on maternal morbidity prior to birth and maternal risk-factors for adverse birth outcomes.[8–12] Whilst existing birth cohort studies across Europe and the US have provided important information on short- and long-term outcomes,[13] they are associated with major costs, are subject to limited numbers for assessing rare conditions, suffer from selectivity in follow-up, and find it difficult to recruit due to increasing participant burden.[14] These limitations provide an imperative for finding alternative approaches using existing data sources. Linked data on a population-level could be used instead for service evaluation and to answer a range of research questions relating to the relationship between pre- and postnatal maternal risk-factors, adverse birth outcomes and healthcare use throughout the course of childhood.[15]

Barriers to linkage between maternal and baby healthcare records in England include uncertainty about the quality of data that are collected primarily for administrative purposes, and the availability and completeness of personal identifiers required for linkage.[15–18] We developed methods for establishing a mother-baby cohort using linkage of a standard, pseudonymised extract of administrative hospital data for England. Our objective was to evaluate the success of mother-baby linkage in the absence of direct personal identifiers, using non-disclosive clinical variables (e.g. dates and delivery information) and demographic variables (e.g. ethnicity and GP practice code). We provide generalisable methods and guidance for combining information on individuals with healthcare characteristics in common.

## Materials and Methods

### Ethics statement

The study is exempt from UK NREC approval because it involved the analysis of an existing dataset of anonymous data for service evaluation. Approvals for the use of HES data were obtained as part of the standard Hospitals Episode Statistics approval process. Hospital Episode Statistics were made available by NHS Digital.

The steps required to create a linked cohort are outlined in the following sections: 1) Understanding the data source; 2) Identifying birth and delivery records; 3) Data preparation; 4) Data linkage; 5) Internal and external validity.

#### 1) Understanding the data source

Data were extracted from Hospital Episode Statistics (HES). HES is an administrative database holding detailed information for all admissions to NHS hospitals in England, and has been collected since 1989, primarily for financial purposes. HES data are made available to researchers with appropriate permissions, in a pseudonymised form (i.e. without personal identifiers) from the HSCIC. Data are divided into financial years, and structured as ‘episodes’ of care, within which a patient is under the care of one consultant. Each admission may comprise multiple episodes; episodes relating to the same individual are assigned the same pseudonymous ID (HESID) by the data provider, allowing researchers to track patient admissions over time without accessing any personal identifiers. HESID is assigned using a deterministic rule-based algorithm based on NHS number, local patient identifier, sex, date of birth and postcode.[19]

HES records contain clinical diagnoses (coded using the International Statistical Classification of Diseases and Related Health Problems 10th Revision: ICD-10[20]), procedures (coded using the Office of Population Censuses and Surveys Classification of Surgical Operations and Procedures 4^th^ revision: OPCS), and Healthcare Resource Groups (HRGs: http://www.hscic.gov.uk/hrg). Standard HES extracts also include sex, month and year of birth and ethnic category (UK census 18 categories). Geographical information includes organisational code (NHS Trust or Primary Care Trust), registered GP practice code, residential postcode district (first 3–4 postcode characters; each postcode district contains an average 9500 UK households) and Index of Multiple Deprivation (IMD, derived from postcode).[21–23]

In addition to the main HES record, delivery episodes for mothers and birth episodes for babies include additional fields called the ‘baby’ (or ‘maternity’) tail.[21] The baby tail contains information on delivery, including gestational age, birth weight and mode of delivery. For multiple births, each delivery record can hold up to 9 baby tails (up to 6 prior to 2002). The baby tail should contain the same information on both maternal and baby records, but is sometimes incomplete.[15] There is no routine linkage of maternal and baby records within HES: the maternal NHS number is not available on the baby record or vice versa.

#### 2) Identifying delivery and birth records

All records relating to birth and delivery episodes for babies and mothers between April 2001 and March 2013 were extracted from HES. Birth episodes can be identified in a number of ways within HES.[15, 18] For this study, maternal (delivery) records were identified by the presence of ICD-10 codes Z37-Z38 (outcome of delivery, liveborn infant), OPCS codes R14-R27 (delivery procedures), or two or more valid baby tail fields (excluding numpreg, numbaby, neocare and well_baby). Baby (birth) records were identified by the presence of ICD-10 codes Z37-Z38, HRG codes N01-N05 (neonates) or HES fields relating to episode type, method of admission, age at start of episode and level of neonatal care. Ectopic pregnancies, terminations and duplicate episodes were excluded. Full descriptions of the code lists are provided in Tables A and B in S1 Appendix.

Maternal records with the same HESID but with episodes <169 days (24 weeks) apart were treated as duplicates (except for multiple births). Baby records with the same HESID, episode start date, start age, postcode district, birth order and birth weight were treated as duplicates. Information on duplicate records was combined and a single record retained. Where multiple births were recorded on the same maternal record, separate delivery records were created for each birth to facilitate linkage with distinct birth records.

The algorithm for assigning HESIDs to multiple episodes of care for the same individual can introduce errors.[24] Where the same HESID was assigned to multiple individuals, records were dropped, as it was not possible to identify the correct record. Where the same individual was assigned multiple HESIDs (i.e. multiple HESIDs for the same episode start date, age, hospital, GP practice, ethnicity, month-year of birth and baby tail fields), records were treated as duplicates and a log was kept of the relevant HESIDs (to facilitate linkage with subsequent episodes of care).

Birth outcomes were identified from clinical information in either baby or maternal records. Multiple births were identified by ICD-10 codes (Z372-Z377 or Z383-Z388), or HES fields ‘birordr’ (birth order) and ‘numbaby’ (number of babies). Preterm births were identified using ‘gestat’ (gestational age) or ICD-10 codes P072, O60 or P590. Still births were identified using three categories of codes: ‘dismeth’ (discharge method), ‘birstat’ (birth status) and ICD-10 diagnosis (see Table C in S1 Appendix for the full code list description).

#### 3) Data preparation

Since postcode is not always completed for birth episodes in HES, postcode was imputed using subsequent episode records up to one year after the birth episode. Where ‘sexbaby’ was not completed for baby records, ‘sex’ as recorded on the main HES record was used.

We hypothesised that any coding errors could occur simultaneously in corresponding maternal and baby records (i.e. if data were input to both records through the same system), and so only minimum data cleaning was applied prior to linkage.[25] Generic values for “not known” or “not applicable” were set to missing. All string variables were trimmed to remove blank characters and instances of “&”, “-”etc were removed (see Table A in S2 Appendix).

#### 4) Data linkage

*Data linkage methods*. There are two main approaches for linking data: deterministic and probabilistic. Deterministic linkage (or rule-based matching) typically requires exact or approximate agreement on a set of common identifiers (e.g. sex, postcode and date of birth). Exact deterministic matching generally achieves few false-matches (where records belonging to different individuals are linked), as it is unlikely that two individuals share the same set of identifiers. However, requiring exact agreement on identifiers can result in low match rates, as any errors or missing values can prevent a match (resulting in missed-matches, where records belonging to the same individual remain unlinked). Deterministic methods can also incorporate approximate matching, e.g. on month and year of birth, phonetic codes or string comparators for names, or dates within a particular timeframe.

Probabilistic linkage is based on deriving a match weight that represents the likelihood of records belonging to the same individual, given the agreement or disagreement on a set of common identifiers.[26] This approach accounts for the discriminative value of each identifier, i.e. agreement on postcode district would contribute more evidence of a match than agreement on sex. Calculation of the match weight depends on the estimation of two conditional probabilities:

- M-probability: the probability that an identifier agrees given records belong to the same individual
- U-probability: the probability that an identifier agrees given records belong to different individuals

The u-probability can be approximated by the probability of chance agreement. For example, the probability of chance agreement on sex is ½. The probability of chance agreement on month of birth is 1/12, and so on. M-probabilities represent the error rate in a particular identifier, and are typically estimated during the linkage process, and updated as more links are made. For example, if sex was miscoded in 5% of record pairs, the m-probability would be 0.95. Frequency-based weights can also be derived, which allows agreement on more rare values to contribute a higher weight.

The overall match weight is derived by calculating the ratio log_2_(m/u) for each identifier, and summing across all identifiers. Record pairs with agreement on multiple identifiers will have large positive match weights; record pairs with disagreement on most identifiers will have negative match weights.

Probabilistic linkage requires cut-off weights to be chosen for classifying record pairs as links or non-links, consequently determining the rates of missed-matches and false-matches. Typically, two thresholds are chosen, and record pairs with weights falling between the thresholds are subjected to further manual review. However, manual review processes can be both subjective and prohibitively time-consuming for large datasets, and often depend on having access to detailed identifying information. An alternative method is to set an optimal error rate (e.g. maximum allowed false-match rate) and to evaluate error rates for a range of threshold values. Estimation of linkage error rates at each potential threshold requires that the true match status is known, and is typically performed using a subset of gold-standard data (e.g. manual review of a sample of records) or by generating synthetic data with similar characteristics to the original data.[27]

*Data linkage methods in this study*. In this study, we firstly used exact deterministic linkage to bring together maternal and baby records, and supplemented this approach with probabilistic linkage of remaining unlinked records (Table 1). To reduce the number of comparison pairs, an initial blocking strategy was employed: mother and baby records were only considered as possible matches if they had been admitted to the same hospital and record pairs with implausible dates were not considered (baby discharged prior to the mother’s admission or mother discharged prior to the baby’s admission). This blocking strategy was subsequently relaxed to capture mothers and babies in different hospitals or where episode dates differed.

**Table 1:** Completeness of potential linkage variables in maternal and baby HES records for 2012/13.

	Potential linkage variable		% Complete (baby extract)	% Complete (maternal extract)	Deterministic linkage	Probabilistic linkage	% agreement in deterministically-linked records^1^
**Main HES record**	Provider code– 3 character	procode3	100.0	100.0	X	X	-
	Code of GP practice	gpprac	93.0	99.6	X	X	-
	Ethnic category	ethnos	92.1	92.6		X*	77.35
	Postcode district of patient residence	postdist	18.0 (38.6)^3^	98.5		X*	97.18
	Date episode started	epistart	100.0	100.0		X*	60.66
	Date episode ended	epiend	100.0	100.0		X*	89.99
	Estimated delivery date^2^	opdte / epistart	99.4	99.5		X*	99.95
	Baby’s age in days	neodur	99.5				
**Baby / Maternity tail**	Sex of baby (or Sex in baby main HES record)	sexbaby	99.9	88.3	X	X	-
	Birth order	birordr	84.6	90.8	X	X	-
	Birth weight	birweit	85.4	89.0	X	X	-
	Length of gestation	gestat	81.7	85.4	X	X*	-
	Mother’s age at delivery	matage	85.3	85.1	X	X	-
	First antenatal assessment date	anasdate	80.9	85.2		X*	99.89
	Gestational period in weeks at first antenatal assessment	anagest	74.6	73.9		X	99.92
	Delivery place (actual)	delplac	87.6	89.1		X	99.93
	Delivery place (intended)	delinten	86.3	88.2		X*	99.22
	Delivery method	delmeth	88.1	88.9		X	99.80
	Method to induce labour	delonset	87.4	89.4		X	99.99
	Anaesthetic given during labour or delivery	delprean	53.9	54.1		X	99.99
	Anaesthetic given post labour or delivery	delposan	26.1	26.8		X	100.00
	Status of person conducting delivery	delstat	85.4	86.9		X*	98.24
	Resuscitation method	biresus	76.5	81.5		X	99.99
	Birth status	birstat	85.1	88.8		X	99.99
	Number of previous pregnancies	numpreg	0.01	71.9			-
	Delivery place change reason	delchang	8.1	7.7			-
	Antenatal days of stay	antedur	86.6	85.2			-
	Postnatal days of stay	postdur	86.9	85.2			-
	Neonatal level of care	neocare	66.6	99.9			-
	Well baby flag	well_baby	100.00	100.0			-
	Number of babies	numbaby	86.9	91.0			-

*Frequency-based probabilistic weights were used for these variables, allowing weights to vary according to the frequency of data values or the distance between dates

^1^ Where fields were complete in both maternity and baby extract;

^2^ Estimated delivery date derived from date of relevant OPCS procedure code (mother) or episode start date (baby);

^3^ Completeness rose to 38.6% when postcode district was imputed from subsequent admission records

Deterministic links were identified as records agreeing exactly on GP practice, maternal age, birth weight, gestation, birth order and sex. Our approach allowed for missing values, as long as at least three of the agreeing variables were complete, and there were no disagreeing values on any variable.

For our probabilistic approach, we used frequency-based match weights since the probability of agreement on a particular variable may vary according to the value of that variable. Frequency weights were derived for each value of gestational age, delivery place (intended), status of person conducting delivery, postcode district (first letter) and ethnic category). For example, this allowed for a higher chance of agreement on a gestational age of 40 weeks (a common value) than 26 weeks (a rare value). Episode start and end dates for mothers and babies could also genuinely be different, e.g. if the mother was admitted the day before delivery. Therefore for dates, match weights were calculated depending on the difference in the number of days (0, 1, 2, 3, 4, 5, 6, 7, 8–14 and 14+ days). Records with dates a small number of days apart would therefore have higher match weights than those that were more than a week apart.

In our study, initial estimates for the m-probabilities were obtained using the deterministically-linked records. U-probabilities were obtained from pairwise comparisons of a random sample of 5000 unlinked records (i.e. 25,000,000 comparisons). Estimates were then iteratively updated using the probabilistically-linked records according to the following steps:

1. Initial match weights were assigned to all record pairs
2. Record pairs were manually reviewed and non-links were removed
3. M-probabilities were re-estimated based on the remaining record pairs
4. New match weights were assigned to all record pairs
5. The process was repeated until match weights stabilised (three iterations in this study)

There is no gold-standard for linkage of maternal and baby records in HES, and even if it were possible to access personal identifiers, maternal and baby HES records do not share a unique identifier. Therefore, linkage quality was evaluated by testing the algorithm and estimating the match rate and false-match rate on synthetic data. Full details of the synthetic data approach are provided in S3 Appendix; in brief, 100 synthetic datasets with similar identifier error rates and missing values to HES were created, where the true match-status was known; after applying the linkage algorithm to each synthetic dataset, false-match rates were estimated.

Completeness of HES fields is known to have improved over time.[15] Therefore, we compared linkage rates for 2001/02 and 2012/13. In order to evaluate the relative contribution to linkage success of more sensitive linkage variables (postcode district and GP practice), we repeated the linkage process excluding these variables.

#### 5) Internal and external validity

We firstly compared values of gestational age and birth weight with published reference values and set to missing values falling more than 3 standard deviations from the average.[28] We then assessed internal validity of maternal and baby records by checking consistency of three rare but important birth outcomes (still births, multiple births and preterm births). Linked maternal-baby records that were discordant on these outcomes were resolved using corroborating information in HES (additional ICD-10 diagnosis codes, the presence of multiple maternity tails, or subsequent admission records).

The representativeness of the linked cohort was evaluated by comparing distributions of key birth characteristics and outcomes with national published data (compiled on birth registrations) from the Office for National Statistics (ONS). Differences were identified using chi^2^ tests for categorical data, t-tests for normal data and the Mann-Whitney U test for skewed data.

## Results

### 1) Understanding the data source and 2) Identifying delivery and birth records

**Fig 1:**
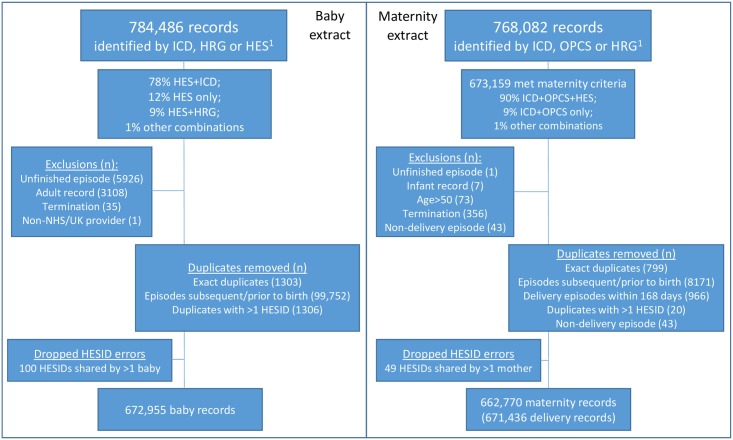
Extract flow-diagram for delivery and birth episodes captured in HES for 2012/13.

The number of records in the baby extract rose from 553,094 in 2001/02 to 672,955 in 2012/13. Fig 1 describes the cohort extraction in detail for 2012/13. HESID assignment errors occurred in <0.01% of maternal records and in up to 0.8% of baby records (Table B in S2 Appendix shows numbers for each year). Completeness of most linkage variables increased over time (Fig A in S2 Appendix).

### 3) Data preparation and 4) Data linkage

Assessment of completeness of clinical and demographic information common to both baby and maternal records showed that fields were generally more well completed for maternity records than baby records (Table 1). Missing postcode was a particular problem for baby records. A number of common variables were not considered for linkage due to missing or non-informative values. For example in maternity records, the Well baby flag always contained the value “N” and the neonatal level of care was almost always “Not applicable”. In baby records, antenatal days of stay was always 0.

For the 2012/13 cohort, 280,237/672,955 baby records (42%) were deterministically linked to a mother. In the deterministically-linked records, agreement on other baby tail variables (those not used in the deterministic linkage) was high (Table 1).

**Fig 2:**
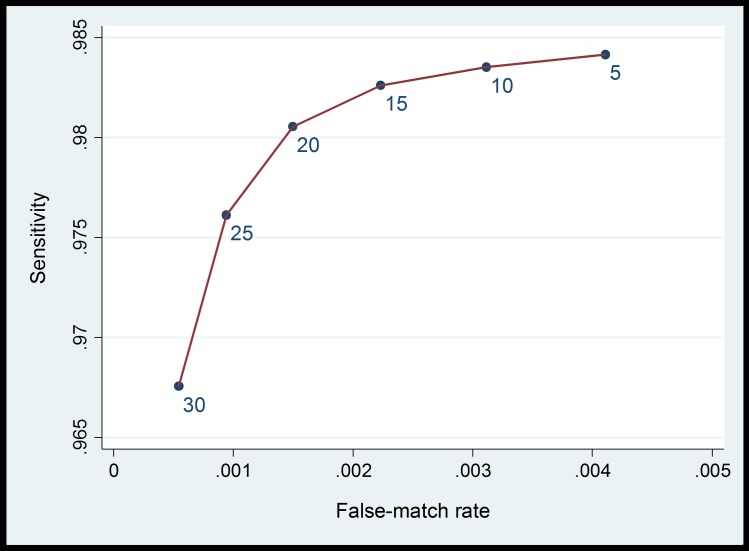
Estimated false-match rate and sensitivity for a range of threshold weights, based on synthetic data.

For probabilistic linkage, final match weights for each linkage variable are provided in Table 2. To choose a threshold, estimates of sensitivity and specificity were derived for combined match weights between 5 and 30, averaged over the 100 synthetic datasets (Fig 2). A threshold of 20 was chosen, for which the false-match rate was estimated as 0.15% in the synthetic data. Probabilistic linkage with this threshold resulted in linkage of a further 380,164 baby records (56%). A total of 660,401 baby records (98%) were therefore linked using deterministic and probabilistic linkage combined.

**Table 2:** Probabilistic match weights.

Linkage variable	HES field name	Match weight	
		Agreement	Disagreement
Sex	sexbaby	0.95	-3.99
GP practice code	gpprac	11.68	-3.07
Maternal age	matage	4.38	-7.40
Birthweight	birweit	8.18	-8.00
Gestational age*	bestat*	2.80	-1.74
Birth order	Birordr	0.04	-7.29
Estimated delivery date^+^	dobbaby^+^	8.48	-10.68
First antenatal assessment date^+^	anasdate^+^	8.37	-3.18
Gestation period in weeks at first antenatal assessment	anagest	3.11	-2.09
Delivery method	delmeth	1.33	-4.21
Delivery place (actual)	delplac	0.94	-1.38
Delivery place (intended)*	delinten*	5.51	-3.50
Method to induce labour	delonset	1.12	-3.20
Anaesthetic given during labour or delivery	delprean	1.77	-4.99
Anaesthetic given post labour or delivery	delposan	1.11	-9.22
Status of person conducting delivery*	delstat*	4.40	-4.77
Birth status	birstat	0.14	-6.10
Resuscitation method	biresus	0.68	-8.55
Ethnic category*	ethnos*	4.26	-1.01
Postcode district of patient residence*	postdist*	10.47	-5.32
Date episode started^+^	epistart^+^	7.79	-1.89
Date episode ended^+^	epiend^+^	8.29	-0.79

*Average of frequency-based weights presented.

^+^Weights presented for date differences of 0 (same day) or 7 days apart.

Accuracy of linkage variables improved over time: more of the baby records with complete values for all deterministic linkage variables matched exactly to a maternal record in 2012/13 than in 2001/02 (78% versus 73%, Table 3). This implies that in 2001/02, at least one variable contained an error in 27% of records, compared with 22% in 2012/13. Linkage of data from 2001/02 had slightly inferior results: 91% of records could be linked whilst retaining the same estimated false-match rate of 0.15%. The highest match rate that could be achieved was 94%, with an associated estimated false-match rate of 1.2%.

**Table 3:** Probability of achieving a deterministic link according to completeness of baby records. The final row shows an increase in accuracy of variables over time: in 2001/02, deterministic links were found for 73.0% of baby records with complete values on all linkage variables compared with 77.5% in 2012/13.

Completeness of deterministic linkage variables						2001/02			2012/13		
GP practice	Maternal age	Birth weight	Gestation	Birth order	Sex of baby	% of all baby records	% deterministically linked	% of all deterministic links	% of all baby records	% deterministically linked	% of all deterministic links
						0.0	0.0	0.0	0.0	0.0	0.0
					✓	1.1	0.0	0.0	1.2	5.0	0.1
				✓	✓	0.0	0.0	0.0	0.0	6.2	0.0
			✓		✓	0.0	0.0	0.0	0.0	20.0	0.0
			✓	✓	✓	0.0	0.0	0.0	0.1	4.0	0.0
		✓			✓	0.0	0.0	0.0	0.0	0.0	0.0
		✓		✓	✓	0.1	0.0	0.0	0.0	8.8	0.0
		✓	✓	✓	✓	1.2	0.2	0.0	0.0	3.9	0.0
	✓				✓	0.0	0.0	0.0	0.0	33.3	0.0
	✓			✓	✓	0.0	1.0	0.0	0.0	39.1	0.0
	✓		✓		✓	0.0	0.0	0.0	0.0	100.0	0.0
	✓		✓	✓		0.0	0.0	0.0	0.0	0.0	0.0
	✓		✓	✓	✓	0.1	0.1	0.0	0.0	58.1	0.1
	✓	✓		✓	✓	0.1	0.4	0.0	0.1	62.1	0.1
	✓	✓	✓		✓	0.0	0.0	0.0	0.0	0.0	0.0
	✓	✓	✓	✓		0.0	0.0	0.0	0.0	100.0	0.0
	✓	✓	✓	✓	✓	4.3	2.9	0.3	1.0	64.1	1.5
✓						0.0	0.0	0.0	0.0	6.8	0.0
✓					✓	9.3	0.0	0.0	31.1	4.4	3.1
✓				✓		0.0	0.0	0.0	0.0	0.0	0.0
✓				✓	✓	0.3	0.0	0.0	0.4	2.4	0.0
✓			✓		✓	1.4	0.0	0.0	1.5	3.1	0.1
✓			✓	✓		0.0	0.0	0.0	0.0	0.0	0.0
✓			✓	✓	✓	0.1	3.2	0.0	1.7	2.7	0.1
✓		✓				0.0	0.0	0.0	0.0	0.0	0.0
✓		✓			✓	0.9	0.0	0.0	0.3	6.1	0.0
✓		✓		✓		0.0	0.0	0.0	0.0	0.0	0.0
✓		✓		✓	✓	2.4	0.0	0.0	0.2	2.2	0.0
✓		✓	✓		✓	0.0	0.0	0.0	0.4	4.3	0.0
✓		✓	✓	✓		0.0	0.0	0.0	0.0	0.0	0.0
✓		✓	✓	✓	✓	18.0	6.5	2.8	2.3	7.4	0.4
✓	✓				✓	0.0	0.0	0.0	0.8	60.8	1.1
✓	✓			✓		0.0	0.0	0.0	0.0	100.0	0.0
✓	✓			✓	✓	0.0	63.8	0.0	0.8	65.0	1.1
✓	✓		✓			0.0	0.0	0.0	0.0	100.0	0.0
✓	✓		✓		✓	0.0	0.0	0.0	0.0	50.9	0.0
✓	✓		✓	✓		0.0	14.3	0.0	0.0	57.1	0.0
✓	✓		✓	✓	✓	2.2	1.7	0.1	4.1	63.5	5.8
✓	✓	✓			✓	0.0	0.0	0.0	0.0	44.4	0.0
✓	✓	✓		✓		0.0	0.0	0.0	0.0	60.0	0.0
✓	✓	✓		✓	✓	4.0	6.6	0.6	9.5	69.8	14.7
✓	✓	✓	✓			0.0	0.0	0.0	0.0	0.0	0.0
✓	✓	✓	✓		✓	2.6	0.0	0.0	0.0	0.0	0.0
✓	✓	✓	✓	✓		0.0	71.4	0.0	0.0	73.3	0.0
✓	✓	✓	✓	✓	✓	51.8	77.5	96.1	44.4	73.0	71.7

**Fig 3:**
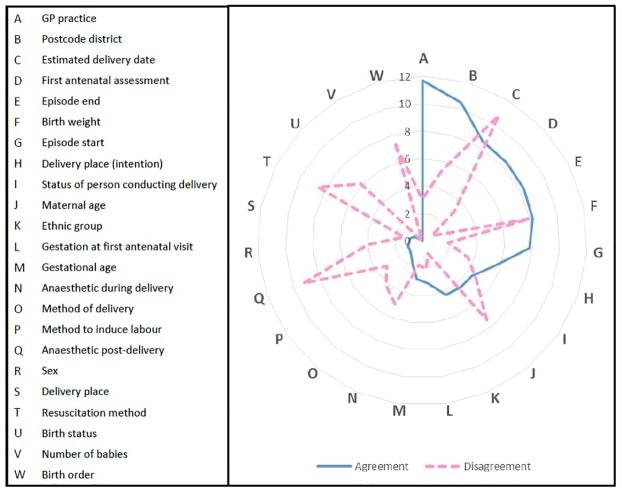
Contribution of each linking variable to overall match weight. Agreement = positive contribution (solid line), disagreement = negative contribution (dashed line). The higher the value, the more information the linkage variable provides.

Some variables were more important than others for linkage (Fig 3). Variables contributing most to the probabilistic linkage (i.e. having highest match weights) were GP practice, postcode district and estimated delivery date (Fig 3, Table 2). Excluding these variables form the linkage process had a detrimental effect: only 80% of baby records could be linked whilst accepting an estimated false-match rate of 0.15%; the highest match rate that could be achieved whilst excluding these variables was 94%, with a corresponding estimated false-match rate of 7%.

It was not possible to link the remaining 2% of baby records, even through manual review of available data. Inspection of the unlinked baby records identified that the main reason for a lack of linkage was missing values: 7452/12,654 (59%) of unlinked records had no baby tail fields compared with 62,157/660,401 (9%) of linked records. Another possible explanation for unlinked baby records is that the mother’s record was not present in the maternal extract (e.g. due to home births where the baby was subsequently admitted but the mother was not). In addition to having more missing values, unlinked records were more likely to be still births, lower gestational age, lower birth weights, younger maternal age, more deprived and non-white but less likely to be multiple births, caesarean sections, or from pregnancies where the first antenatal assessment was before 20 weeks (Table 4).

**Table 4:** Comparison of linked and unlinked baby record characteristics for 2012/13. Missing values are excluded from all categories.

		Linked N = 660,401	% 98%	Unlinked N = 12,654	% 2%	p-value
**Missing all baby tail fields**		62157	**9.4**	7452	**58.9**	<0.001
**Maternal age (years)**	*Mean*	*29*.*3*		*29*.*6*		0.003
	< = 18	11452	2.6	103	2.9	
	19–24	87116	20.0	793	22.0	
	25–29	120943	27.7	1036	28.8	
	30–34	129398	29.7	898	24.9	
	35–39	68916	15.8	519	14.4	
	> = 40	18008	4.1	252	7.0	
IMD quintile*	Most deprived	9269	19.7	1107	22.2	<0.001
	2	9410	20.0	970	19.5	
	3	9388	20.0	1000	20.1	
	4	9380	20.0	1012	20.3	
	Least deprived	9491	20.2	894	17.9	
**Ethnicity**	White	457181	75.2	7855	71.0	<0.001
	Mixed	29234	4.8	693	6.3	
	Asian	68588	11.3	1219	11.0	
	Black	32410	5.3	735	6.6	
	Other	20299	3.3	567	5.1	
**Gestational age (weeks)**	*Median*	*40*		*39*		<0.001
	< = 27	5674	1.0	174	4.4	
	28-<32	6591	1.2	84	2.1	
	32-<37	31872	5.8	348	8.8	
	37-<42	478042	87.5	3229	81.3	
	> = 42	23881	4.4	137	3.4	
**Birth weight (g)**	*Mean*	*3342*		*3106*		<0.001
	<1000	2837	0.5	159	3.4	
	1000–1499	3516	0.6	116	2.5	
	1500–2499	33021	5.8	498	10.6	
	2500–3999	465984	81.7	3513	74.6	
	> = 4000	64710	11.4	425	9.0	
**First antenatal assessment <20 weeks**		443262	88.8	2508	86.7	<0.001
**Caesarean section**		146077	24.8	787	17.1	<0.001
**Still birth**		2752	0.4	285	2.3	<0.001
**Multiple birth**		20436	3.1	352	2.8	0.041

*IMD = Index of Multiple Deprivation

### 5) Internal and external validity

**Fig 4:**
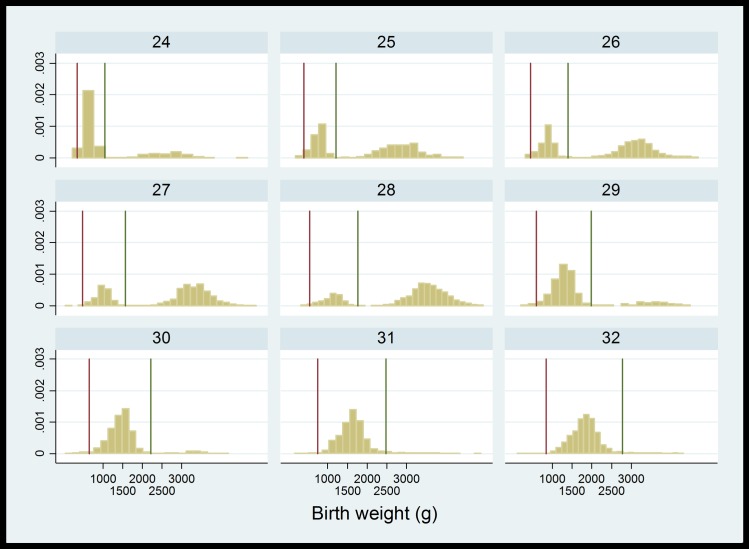
Distribution of birth weight by week of gestation in baby records. Vertical lines show 3 standard deviations from the average; values above the upper limit are likely to have been miscoded as days (rather than weeks) of gestation, truncated to 2 digits.

Inspection of the linked cohort in terms of gestational age and birthweight distributions identified coding issues specific to individual hospital providers. For example, one hospital coded gestational age in days rather than weeks (e.g. 280 days rather than 40 weeks). As gestational age was truncated at two digits, this meant that the majority of babies within this hospital appeared to be born preterm with unfeasibly large birthweight (Fig 4). Similarly, birthweight was truncated at 2 or 3 digits for a small number of records, indicating weights recorded as kilograms rather than grams.

### Internal validity

#### Preterm birth

Gestational age was available for 546,083/660,401 (83%) linked baby records and 567,699/660,401 (86%) linked maternal records for 2012/13. Completeness of gestational age increased to 92% when using information from either record. Gestational age was discordant on 4% of linked baby-maternal records, and the majority of these (71%) differed by 1 or 2 weeks only. For discordant records, the value in the maternal record seemed to be more accurate (based on birth weight for gestational age). Only 4 records had an ICD-10 code for preterm birth but a gestational age >37 weeks.

#### Multiple births

Multiple birth status was discordant in 1860/660,401 (0.3%) record pairs, suggesting missing or inaccurate records. For 617 pairs, there was evidence of a multiple birth in the maternal record but not in the baby record. For 1243 pairs, there was evidence of multiple birth in the baby record, but only one maternal record.

#### Still births

Still birth status was discordant in 1232/660,401 (0.2%) record pairs. For 1165 pairs, still birth was recorded in the maternal record but not the baby record. For 67 pairs, stillbirth was recorded in the baby record but not the maternal record. Discordant records were resolved by checking the baby’s length of stay: if length of stay was >1 day, still births were reclassified as live births. The majority of these errors were related to multiple births: maternal records with ICD10 code Z373 (Twins, one liveborn and one stillborn) or birth status in the maternity record baby tail.

### External validity

**Fig 5:**
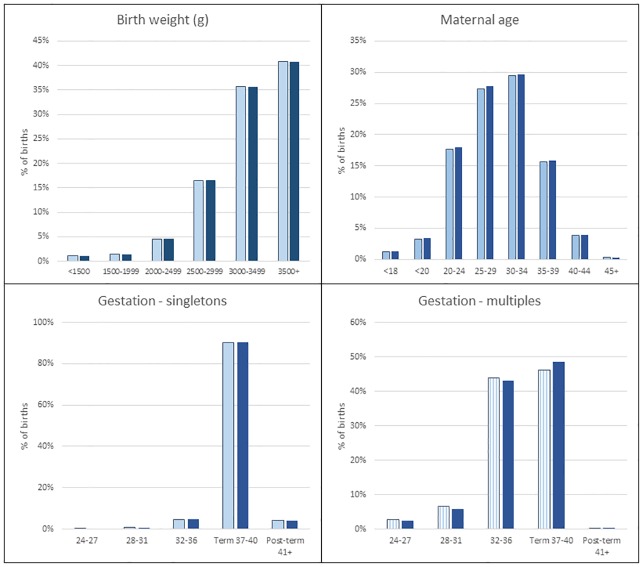
Representativeness of linked HES cohort in terms of maternal age, birth weight and gestational age. Dark shade = HES, light shade = Office for National Statistics.

The linked birth cohort captured 660,401 births for 2012/13 (equating to 97% of total births in English hospitals according to the ONS) and was representative of national data in terms of distributions of gender (51.3% males in both data sources), gestational age, birth weight and maternal age (Fig 5). Although babies with adverse outcomes (lower gestational age, lower birth weight etc) were less likely to be linked, the absolute number of records that failed to link from these groups was low. Overall, there were no differences in any of the key characteristics or birth outcomes between ONS data and the linked cohort: still birth rates were 0.49% (ONS) and 0.54% (linked cohort); multiple birth rates were 3.17% (ONS) and 3.09% (linked cohort); preterm birth rates were 7.09% (ONS) and 7.29% (linked cohort).

## Discussion

### Main findings

Our study demonstrates the feasibility of linking maternal and baby healthcare characteristics using a range of clinical and demographic variables captured in pseudonymised hospital data. We demonstrate a linkage approach that can be used to enhance information health on electronic health records but that does not require the release of any personal identifiers and therefore preserves existing levels of confidentiality within the data. Triangulating outcomes recorded in different hospital records can help improve data quality. Our methods are generalisable to linkage of administrative data in other contexts, where all available information can be combined into “indirect” identifiers for linkage.[29]

The main limitation of linking administrative or electronic healthcare data is the imperfect nature of data collected for reasons other than research.[30] Compared with data collection in a busy healthcare environment, research studies often have more capacity for quality control, for example a birth cohort study or rolling survey is likely to be more complete due to more opportunities for validation, and a greater level of importance given to the accuracy of variables collected.[30] Furthermore, discordance within and between maternal and baby records in our study indicates that there remains uncertainty in coding of some conditions or events. However, our study also demonstrates that linkage can be used to generate high quality data, through triangulating outcomes coded in different hospital records, and improving ascertainment of outcomes by combining information from different sources.

We also demonstrate that validation of data quality using external sources (such as national birth registration data from ONS) can support the use of these data for specific purposes but also helps to highlight where limitations in the data lie. For example in this linked dataset, there remained some uncertainty about coding of still births within multiple birth pregnancies. The implications of any uncertainty or inconsistencies in coding or potential selection bias need to be carefully considered in light of the proposed use for the data. Quality of linked data should be carefully reported, e.g. by comparing characteristics of linked and unlinked records to identify potential sources of bias, so that researchers and policy makers can assess the relevance of the resulting data for their purposes.[30–32]

Errors occurring during linkage (missed-matches and false matches) can result in substantially biased results: false-matches can bias associations towards the null and missed-matches can lead to selection bias.[31, 33] Our evaluation of linkage quality supports evidence from other studies showing differing data quality between subgroups, as more babies at extremes of birth weight and gestational age remained unlinked.[33] However, probabilistic linkage produced a large sample of linked records (660,401: 97% of babies born in 2012/13) and comparisons with published data indicated that the linked data were nationally representative in terms of key birth characteristics and outcomes. Although there is no gold-standard for evaluating linkage quality for HES, and it was not possible to access personal identifiers to perform detailed manual review, synthetic data provide a convenient method for estimating false-match rates. The estimated false-match rate of 0.15% was unlikely to introduce any substantial bias into the linked data. Where this is not the case, statistical methods such as imputation can be considered to account for bias due to linkage error.[31, 34, 35]

In exploiting individual-level data for public benefit, data providers and data users have a responsibility both to ensure that confidential information is protected, and that the data are as accurate as possible. There is a growing body of literature on data confidentiality, some of which argues that individual-level data can never be truly anonymous, depending on external information available to individuals accessing that data.[36] However, there are a number of safeguards in place to protect against inadvertent misuse of data, and restrict the ability of any individual to purposefully behave in a way that jeopardizes data security. Firstly researchers have a responsibility to use data for bona fide purposes only, and there are legal sanctions where data are used inappropriately or without due care. Data access approval processes require that researchers be regularly trained in information governance, to avoid any accidental data breaches. Secondly, secure physical locations (known as safe havens or safe pods) have been established for the processing and linkage of personal data, and are characterised by strict access arrangements, secure data transfer processes, restricted network and/or internet access, and tight disclosure control procedures.[36] Whilst using direct personal identifiers for this study could have helped achieve the highest level of accuracy in the resulting data, restricting the release of personal identifiers provides further protection against outsiders with malicious intent. In the context of current information governance and data protection regulations in the UK, researchers can very rarely access personal identifiers and more innovative linkage methods, such as those used in our study, are required.

Although our study only combined information for mothers and babies relating to the same admission (the delivery / birth episode), the longitudinal nature of HES allows admissions for the same individual to be linked over time. This means that in addition to enriching maternity data, this linkage provides an opportunity for evaluating how pre- and postnatal maternal medical histories (that are solely captured in maternal records) influence infant and childhood outcomes.[37] Such data are particularly useful for investigating the effect of exposures during pregnancy on outcomes throughout childhood, and could be enhanced further through linkage to different sources of data such as primary care and education. Linkage of retrospective electronic healthcare data can be useful for resolving data quality issues, and could be used to supplement evidence from cohort studies and prospective data collection such as the HSCIC maternity and children’s dataset. Ultimately, these data will improve our understanding of maternal risk factors for childhood outcomes, e.g. for assessing the effects of prenatal exposure to drugs or maternal mental health.[38, 39] Given appropriate safeguards, linked maternal-baby data could be made available as a resource for service evaluation and research, to complement linkage of prospective maternity and child health datasets in the UK.

### Conclusions

Probabilistic linkage of maternal and baby healthcare characteristics offers an efficient way to enrich maternity data, improve data quality, and create longitudinal cohorts for research and service evaluation, without the use of direct patient identifiers. Combining information from multiple sources can help to address data quality issues in electronic health data, and the approaches described here could be extended to other administrative data sources. Linked maternal-baby hospital records in England provide a nationally representative resource for service evaluation and research on the impact of maternal risk-factors and interventions on outcomes in childhood.

## Supporting Information

S1 Appendix Identifying delivery and birth records. (DOCX)

S2 Appendix Data preparation. (DOCX)

S3 Appendix Data linkage. (DOCX)
