# Supplementary material for: Linking Data for Mothers and Babies in De-Identified Electronic Health Data
Source: PLoS One. 2016 Oct 20;11(10):e0164667. doi: 10.1371/journal.pone.0164667 (PMC5072610; doi:10.1371/journal.pone.0164667)
Supplement: S1 Appendix — (DOCX) [file pone.0164667.s001.docx]

# **S1 Appendix: Identifying delivery and birth records**

##### **S1 Table A: Code list used for identifying maternity episodes in HES.**

|  | **Codeset** | **Value** | **Description** |
| --- | --- | --- | --- |
| **Inclusion** | **OPCS** | R14 | Surgical induction of labour |
|  |  | R15 | Other induction of labour |
|  |  | R17 | Elective caesarean delivery |
|  |  | R18 | Other caesarean delivery |
|  |  | R19 | Breech extraction delivery |
|  |  | R20 | Other breech delivery |
|  |  | R21 | Forceps cephalic delivery |
|  |  | R22 | Vacuum delivery |
|  |  | R23 | Cephalic vaginal delivery with abnormal presentation of head |
|  |  | R24 | Normal delivery |
|  |  | R25 | Other methods of delivery |
|  |  | R27 | Other operations to facilitate delivery |
|  | **HES**  **(at least two valid values required)** | delprean | Anaesthetic given during labour or delivery |
|  |  | delposn | Anaesthetic given post-labour or delivery |
|  |  | antedur | Antenatal days of stay |
|  |  | dobbaby | Birth date (baby) |
|  |  | birorder | Birth order |
|  |  | birweit | Birthweight |
|  |  | delchang | Delivery place change reason |
|  |  | delmeth | Delivery method |
|  |  | delplac | Delivery place (actual) |
|  |  | delinten | Delivery place (intended) |
|  |  | anasdate | First antenatal assessment date |
|  |  | anagest | Gestation period in weeks at first antenatal assessment |
|  |  | gestat | Length of gestation |
|  |  | birstat | Birth status |
|  |  | delonset | Labour / delivery onset method |
|  |  | matage | Mother’s age at delivery |
|  |  | postdur | Postnatal stay |
|  |  | biresus | Resuscitation method |
|  |  | sexbaby | Sex of baby |
|  |  | delstat | Status of person conducting delivery |
|  | **ICD-10** | Z37. | Outcome of delivery |
|  |  | Z38. | Liveborn infants according to place of birth |
| **Exclusion** | **HES** | epistat = 1 | Unfinished episode |
|  |  | startage>7000  startage<12 | Infant  Infant |
|  |  | startage>50 | Unlikely mother |
|  |  | epiend = . | Missing episode end date |
|  |  | procode begins with N / 8 | Non-NHS or non-UK provider |
|  |  | gestat <24 | Gestational age at birth <24 weeks |
|  | **ICD10** | O00 | Ectopic pregnancy |
|  |  | O01 | Hydatidiform mole |
|  |  | O02 | Other abnormal products of conception |
|  |  | O03 | Spontaneous abortion |
|  |  | O04 | Medical abortion |
|  |  | O05 | Other abortion |
|  |  | O06 | Unspecified abortion |
|  |  | O07 | Failed attempted abortion |
|  |  | O08 | Complications following abortion / ectopic / molar pregnancy |
|  |  | P964 | Termination of pregnancy, affecting fetus and newborn |

##### **S1 Table B: Code list for identifying birth episodes in HES. Records were defined as birth episode if any of the criteria were met.**

| **Codeset** | **Value** | **Description** |
| --- | --- | --- |
| **ICD10** | Z37 | Outcome of delivery |
|  | Z38 | Live born infant |
| **HES** | epitype | 3: Birth event |
|  |  | 6: Other birth event |
|  | admimeth | 82: Other: babies born in health care provider |
|  |  | 83: Other: babies born outside the health care provider, except when born at home as intended |
|  | startage | 7001: <1 day |
|  |  | 7002: 1-6 days |
|  | neocare | 0: Normal care |
|  |  | 1: Special care |
|  |  | 2: Level 2 intensive care |
|  |  | 3: Level 1 intensive care |
| **HRG** | N01 | Neonates – died <2 days old |
|  | N02 | Neonates with multiple minor diagnoses |
|  | N03 | Neonates with one minor diagnosis |
|  | N04 | Neonates with multiple major diagnoses |
|  | N05 | Neonates with one major diagnosis |

##### **S1 Table C: Code list for identifying birth outcomes in HES.**

| **Outcome** | **Codeset** | **Value** | **Description** |
| --- | --- | --- | --- |
| **Multiple birth** | ICD10 | Z372 | Twins, both liveborn |
|  |  | Z373 | Twins, one liveborn and one stillborn |
|  |  | Z374 | Twins, both stillborn |
|  |  | Z375 | Other multiple births, all liveborn |
|  |  | Z376 | Other multiple births, some liveborn |
|  |  | Z377 | Other multiple births, all stillborn |
|  |  | Z383 | Twin, born in hospital |
|  |  | Z384 | Twin, born outside hospital |
|  |  | Z385 | Twin, unspecified as to place of birth |
|  |  | Z386 | Other multiple, born in hospital |
|  |  | Z387 | Other multiple, born outside hospital |
|  |  | Z388 | Other multiple, unspecified as to place of birth |
|  | HES | birordr>1 | Birth order |
|  |  | numbaby>1 | Number of babies |
| **Preterm birth** | ICD10 | P072 | Extreme immaturity |
|  |  | O60 | Preterm labour and delivery |
|  |  | P590 | Neonatal jaundice associated with preterm delivery |
|  | HES | gestat<37 | Length of gestation |
| **Still birth** | ICD10 | P95 | Fetal death of unspecified cause |
|  |  | Z371 | Single stillbirth |
|  |  | Z373 | Twins, one liveborn and one stillborn |
|  |  | Z374 | Twins, both stillborn |
|  |  | Z376 | Other multiple births, some liveborn |
|  |  | Z377 | Other multiple births, all stillborn |
|  | HES | birstat=2-4 | Birth status |
|  |  | dismeth=5 | Method of discharge |
