# Supplementary material for: Linking Data for Mothers and Babies in De-Identified Electronic Health Data
Source: PLoS One. 2016 Oct 20;11(10):e0164667. doi: 10.1371/journal.pone.0164667 (PMC5072610; doi:10.1371/journal.pone.0164667)
Supplement: S2 Appendix — (DOCX) [file pone.0164667.s002.docx]

# **S2 Appendix: Data preparation**

#### **S2 Table A: Data cleaning prior to linkage**

| **Variable** | **Variable name** | **Values set to null*** |
| --- | --- | --- |
| Episode start date | epistart | <= 01/01/1600 🡪 drop record |
| Episode end date | epiend | <= 01/01/1600 🡪 drop record |
| Month and year of birth | mydob | <=01/01/1901 |
| Age at start of episode | startage | If mydob is null, startage 🡪 null |
| Sex of baby | sexbaby | M🡪1; F🡪2  Any other value 🡪 null |
| Birthweight | birweit | >7000 |
| Gestational age | gestat | 99 |
| Birth status | birstat | 9 |
| Resuscitation method | biresus | 9 |
| Delivery place (actual) | delplac | 9 |
| Status of person conducting delivery | delstat | 9 |
| Birth order | birordr | 8, 9 |
| Delivery place (intended) | delinten | 9 |
| Method to induce labour | delonset | 8, 9 |
| Anaesthetic given during labour or delivery | delprean | 8, 9 |
| Anaesthetic given post labour or delivery | delposan | 8, 9 |
| Number of babies | numbaby | >9 |
| Mother’s age at delivery | matage | 0, >100 |
| Birth date (baby) | dobbaby | <01/01/1996  >31/03/2014 |

*unless otherwise stated

## **S2 Table B: Number of records extracted by year and number of HESID assignment errors within maternal and baby extracts.** Type 1 errors = the same HESID assigned to two different individuals: at least two records shared the same HESID but different birth weights. Type 2 errors = the same individual was assigned multiple HESIDs: at least two records shared the same episode start date, age, hospital, GP practice, ethnicity, month-year of birth, and baby tail fields but had different HESIDs.

|  | **Maternal cohort** | | | | | | | **Baby cohort** | | | | | |
| --- | --- | --- | --- | --- | --- | --- | --- | --- | --- | --- | --- | --- | --- |
|  | **Deliveries** | **Maternal records** | **Unique HESIDs** | **N HESIDs shared by >1 mother** | **Type 1 error rate (%)** | **N mothers with**  **>1 HESID** | **Type 2 error rate (%)** | **Birth records** | **Unique HESIDs** | **N HESIDs shared by >1 baby** | **Type 1 error rate (%)** | **N babies with**  **>1 HESID** | **Type 2 error rate (%)** |
| **2001/02** | 527,514 | 521,829 | 521,486 | 20 | 0.00% | 3 | 0.00% | 553,094 | 553,017 | 76 | 0.01% | 2334 | 0.42% |
| **2002/03** | 540,071 | 535,267 | 534,884 | 26 | 0.00% | 8 | 0.00% | 563,695 | 563,616 | 76 | 0.01% | 2266 | 0.40% |
| **2003/04** | 570,607 | 565,459 | 565,042 | 35 | 0.01% | 3 | 0.00% | 578,695 | 578,570 | 115 | 0.02% | 2487 | 0.43% |
| **2004/05** | 578,019 | 572,174 | 571,733 | 42 | 0.01% | 5 | 0.00% | 592,842 | 592,752 | 89 | 0.02% | 2443 | 0.41% |
| **2005/06** | 593,465 | 587,771 | 587,276 | 32 | 0.01% | 9 | 0.00% | 603,416 | 603,324 | 89 | 0.01% | 2308 | 0.38% |
| **2006/07** | 611,922 | 605,245 | 604,805 | 39 | 0.01% | 7 | 0.00% | 618,377 | 618,249 | 124 | 0.02% | 2386 | 0.39% |
| **2007/08** | 634,249 | 629,971 | 629,485 | 40 | 0.01% | 8 | 0.00% | 638,189 | 638,055 | 132 | 0.02% | 2722 | 0.43% |
| **2008/09** | 647,408 | 640,268 | 639,774 | 58 | 0.01% | 2 | 0.00% | 650,275 | 650,154 | 120 | 0.02% | 1558 | 0.24% |
| **2009/10** | 653,364 | 644,337 | 643,847 | 60 | 0.01% | 1 | 0.00% | 661,535 | 661,385 | 147 | 0.02% | 1061 | 0.16% |
| **2010/11** | 669,435 | 660,714 | 660,259 | 51 | 0.01% | 2 | 0.00% | 673,495 | 673,339 | 151 | 0.02% | 1536 | 0.23% |
| **2011/12** | 674,208 | 665,187 | 664,683 | 70 | 0.01% | 9 | 0.00% | 680,219 | 680,065 | 152 | 0.02% | 1089 | 0.16% |
| **2012/13** | 671,436 | 662,770 | 662,305 | 49 | 0.01% | 20 | 0.00% | 672,955 | 672,949 | 100 | 0.01% | 1251 | 0.19% |

## **S2 Fig A: Completeness of linking variables in maternal (delivery) records and birth (baby) records.** Dashed lines = maternity record; solid lines = baby record.
