# Supplementary material for: Linking Data for Mothers and Babies in De-Identified Electronic Health Data
Source: PLoS One. 2016 Oct 20;11(10):e0164667. doi: 10.1371/journal.pone.0164667 (PMC5072610; doi:10.1371/journal.pone.0164667)
Supplement: S3 Appendix — (DOCX) [file pone.0164667.s003.docx]

# **S3 Appendix: Data linkage**

## **Synthetic data to choose the cut-off threshold and evaluate linkage quality**

After final match weights had been calculated, cut-off threshold weights are required for classifying record pairs as links or non-links. For this purpose, we created synthetic data from which to estimate false-match and missed-match rates at a number of different thresholds.

Following deterministic linkage and blocking on hospital and dates, there were 312,287 candidate record pairs remaining in 2012/13. Manual inspection of these pairs suggested that a broad range for appropriate thresholds was between match weights of 10 and 25. This indicated that the proportion of baby records that could match a maternal record was between 98.3-99.6%.

To create a synthetic set of candidate record pairs with similar properties, we firstly calculated identifier error/missing value rates in records with match weights >10 and >25 respectively. For example, the identifier error rate for birth weight was estimated as 12.6% for pairs with a match weight >10 (12.6% of matched records differed on birth weight) or 12.1% for pairs with a match weight >25. The percentage of record pairs agreeing by chance on birthweight was similarly estimated, at between 9.2% and 10.5%.

The synthetic data were then created in the following stages:

1. A synthetic baby extract was created by randomly sampling values of each linking variable from the baby extract.
2. The proportion of these baby records with a matching maternal record was randomly drawn from the range 98.3-99.6% (range chosen on inspection of the candidate record pairs as described above).
3. For each baby record, a corresponding candidate maternal record was created by taking an exact copy of the baby record.
4. Errors and missing values were introduced using error rates derived from manual inspection of the candidate record pairs, e.g.:

- for ‘true’ matches, between 12.1-12.6% of birth weight values in the maternal record were altered
- for ‘false’ matches, between 9.2-10.5% of birth weight values were allowed to agree by chance in the maternal record

1. Match weights were assigned to each candidate record pair
2. Steps were repeated to create 100 versions of the synthetic dataset.

Since the true match status was known in the synthetic data, it was possible to directly count the number of false-matches and missed-matches at each cut-off threshold. Estimates of sensitivity and specificity were derived for match weights between 5 and 30, averaged over the 100 synthetic datasets (Figure 4). We chose a threshold of 20, for which the estimated false-match rate was 0.15% and the sensitivity was 98.1%. This threshold provided a good balance between sensitivity and specificity.
